# Supplementary material for: Meta-programmable analog differentiator
Source: Nat Commun. 2022 Mar 31;13:1713. doi: 10.1038/s41467-022-29354-w (PMC8971527; doi:10.1038/s41467-022-29354-w)
Supplement: Supplementary file 1 — Supplementary Information [file 41467_2022_29354_MOESM1_ESM.pdf]

Supplementary Materials for

Meta-Programmable Analog Differentiator

Jérôme Sol[1], David R. Smith[2], and Philipp del Hougne[3*]

[1] INSA Rennes, CNRS, IETR - UMR 6164, F-35000, Rennes, France

[2] Center for Metamaterials and Integrated Plasmonics, Department of Electrical and Computer Engineering, Duke University, Durham, NC, 27708 USA

[3] Univ Rennes, CNRS, IETR - UMR 6164, F-35000, Rennes, France

* Correspondence to philipp.del-hougne@univ-rennes1.fr.

Table of Contents:

Supplementary Note 1: Remarks on Coherent Perfect Absorption

Supplementary Note 2: Transfer Function of an Ideal Differentiator

Supplementary Note 3: Vulnerability of Analog Differentiators

Supplementary Note 4: Details on the Experimental Setup

Supplementary Note 5: Details on the Experimental Procedure

Supplementary Note 6: Experimentally Measured Notch Depths

Supplementary Note 7: Error Performance Evaluation: Bandwidth and Dip Symmetry

Supplementary Note 8: Operation in Transmission Mode

Supplementary Note 9: Further Examples of Parallel Computing

Supplementary Note 10: Generalizations

Supplementary References

Supplementary Note 1. Remarks on Coherent Perfect Absorption

The following remarks complement the discussion in the introduction of our main text:

A. Nature of the loss mechanism

Because coherent perfect absorption (CPA) has been introduced as the time-reverse of lasing (at threshold)[1], the loss mechanisms should originate from matter susceptibility rather than from undetected elastic scattering. Recently, there has been a growing interest in reflectionless coupling into (often quasi-lossless) structures which let radiation escape through unobserved channels[2–4]. Sometimes this has also been referred to as CPA[5] even though in these cases wave energy is *not* irreversibly transduced into other degrees of freedom such as heat. In the experiments we present, losses originate from absorption at the cavity boundaries (in line with the original definition of CPA), but the loss mechanism's nature is irrelevant for our general idea.

B. Dwell time divergence

The divergence of the dwell time can also be understood from considering the *phase delay time* which is defined as the frequency derivative of the scattering phase: $\tau = \mathrm{d}(\arg(S_{11}))/\mathrm{d}\omega$.[6] Given the discontinuity of the phase of the transfer function $i(\omega - \omega_0)$ at $\omega = \omega_0$, namely its phase jump of π, its derivative at $\omega = \omega_0$ diverges.

C. Tuneable microwave notch filters

Tunable microwave notch filters are developed for applications other than analog differentiation where different metrics matter, resulting in filter shapes that are typically not only insufficiently deep but also oftentimes deformed with respect to the shape needed for analog differentiation. In other words, tunable microwave notch filters do not implement *perfect* absorption.

Supplementary Note 2. Transfer Function of an Ideal Differentiator

In this supplementary note, we prove for completeness that the transfer function of an ideal differentiator is $H(\omega) = i(\omega - \omega_0)$. We also discuss the impact of an additional background phase drift with frequency on the filter's functionality. Finally, we provide the transfer function of an ideal second-order differentiator.

Let $e(t)$ be the function whose derivative is to be determined. This function $e(t)$ modulates a carrier signal of angular frequency ω_0, yielding the input signal

$$E_{in}(t) = e(t)e^{i\omega_0 t}.$$

The desired output signal is thus

$$E_{out}(t) = \frac{\mathrm{d}e(t)}{\mathrm{d}t}e^{i\omega_0 t}.$$

The Fourier transform of $E_{out}(t)$ is

$$\tilde{E}_{out}(\omega) = \int_{-\infty}^{\infty} \frac{\mathrm{d}e(t)}{\mathrm{d}t} e^{-i(\omega-\omega_0)t}\mathrm{d}t.$$

Integration by parts yields

$$\tilde{E}_{out}(\omega) = e^{-i(\omega-\omega_0)t}e(t)\Big|_{-\infty}^{\infty} + i(\omega - \omega_0)\int_{-\infty}^{\infty} e(t)\, e^{-i(\omega-\omega_0)t}\mathrm{d}t = i(\omega - \omega_0)\tilde{E}_{in}(\omega),$$

where we assume that $\lim_{t\to\pm\infty} e(t) = 0$ for any realistic signal $e(t)$.

The transfer function associated with the differentiation operation is thus

$$H(\omega) = \frac{\tilde{E}_{out}(\omega)}{\tilde{E}_{in}(\omega)} = i(\omega - \omega_0).$$

An analog differentiator is in general satisfactory if its output is proportional (not necessarily equal) to $E_{out}(t)$, i.e., $H(\omega) \propto i(\omega - \omega_0)$ is needed. In practice, we observe that instead we achieve $H(\omega) \propto i(\omega - \omega_0)e^{-i\tau\omega}$ due to a slow background phase drift with frequency. The basic time-shifting property of the Fourier transform, namely that the Fourier transform of $X(t - t_0)$ is $e^{-i\omega t_0}\tilde{X}(\omega)$, implies that this background phase drift $e^{-i\tau\omega}$ just shifts the desired output signal in time from $E_{out}(t)$ to $E_{out}(t - \tau)$, and hence poses no problem for the analog differentiator.

The transfer function of higher-order derivatives is simply the product of several first-order differentiator transfer functions, e.g., for the second derivative, $\frac{\mathrm{d}^2y}{\mathrm{d}t^2} = \frac{\mathrm{d}}{\mathrm{d}t}\frac{\mathrm{d}y}{\mathrm{d}t}$, the corresponding transfer function is $|H(\omega)|^2 = -(\omega - \omega_0)^2$. The phase of an ideal second-order differentiator is thus perfectly flat.

Supplementary Note 3. Vulnerability of Analog Differentiators

In this supplementary note, we illustrate the vulnerability of analog differentiators in order to highlight why it is important to *perfectly* implement the transfer function derived in Supplementary Note 2 in the experiment. Specifically, we show that an experimental setup does not faithfully act as differentiator if it

(i) relies on a well-matched port (e.g., at $-30\,\mathrm{dB}$) as opposed to a perfectly matched port,

(ii) gets the central operating frequency slightly wrong, or

(iii) presents an asymmetry around the central frequency.

To substantiate these points, without loss of generality, we take the analytical transfer function of a differentiator based on a simple Mach-Zehnder interferometer (MZI, akin to Refs.[7,8]) as starting point in order to work with a physically justified analytical expression. Assuming that transmission through the upper MZI arm, relative to the lower MZI arm, introduces an additional time delay τ and differs in amplitude by a factor A, then the transfer function reads $H(\omega) = 1 + Ae^{i\omega\tau}$. Under idealistic conditions, $A = 1$ and one would choose $\tau = \frac{(2m+1)\pi}{\omega_0}$, where m is an arbitrary integer, such that $H(\omega_0) = 0$ and the desired transfer function from Supplementary Note 2 would be obtained in the vicinity of ω_0 (see Refs.[7,8]).

In column **a** of Supplementary Figure 1, we depict the ideal case in which the transfer function perfectly matches the required one (see Supplementary Note 2) in terms of magnitude and phase, and the output signal upon injection of a Gaussian envelope is perfectly symmetric and has exactly zero amplitude at its center.

In column **b**, we introduce a minimal error by setting $A = 0.9975$ instead of unity. The magnitude of the transfer function thereby deteriorates to $-33.7\,\mathrm{dB}$ at ω_0 which is not even visible on the linear magnitude plot in the first row. However, it is clearly visible that the phase jump is not abrupt anymore. The output signal already displays substantial distortions: it is not symmetric anymore in the time domain and there is a non-zero component at ω_0 in its spectrum. This highlights that a well-matched port ($-33.7\,\mathrm{dB}$) is

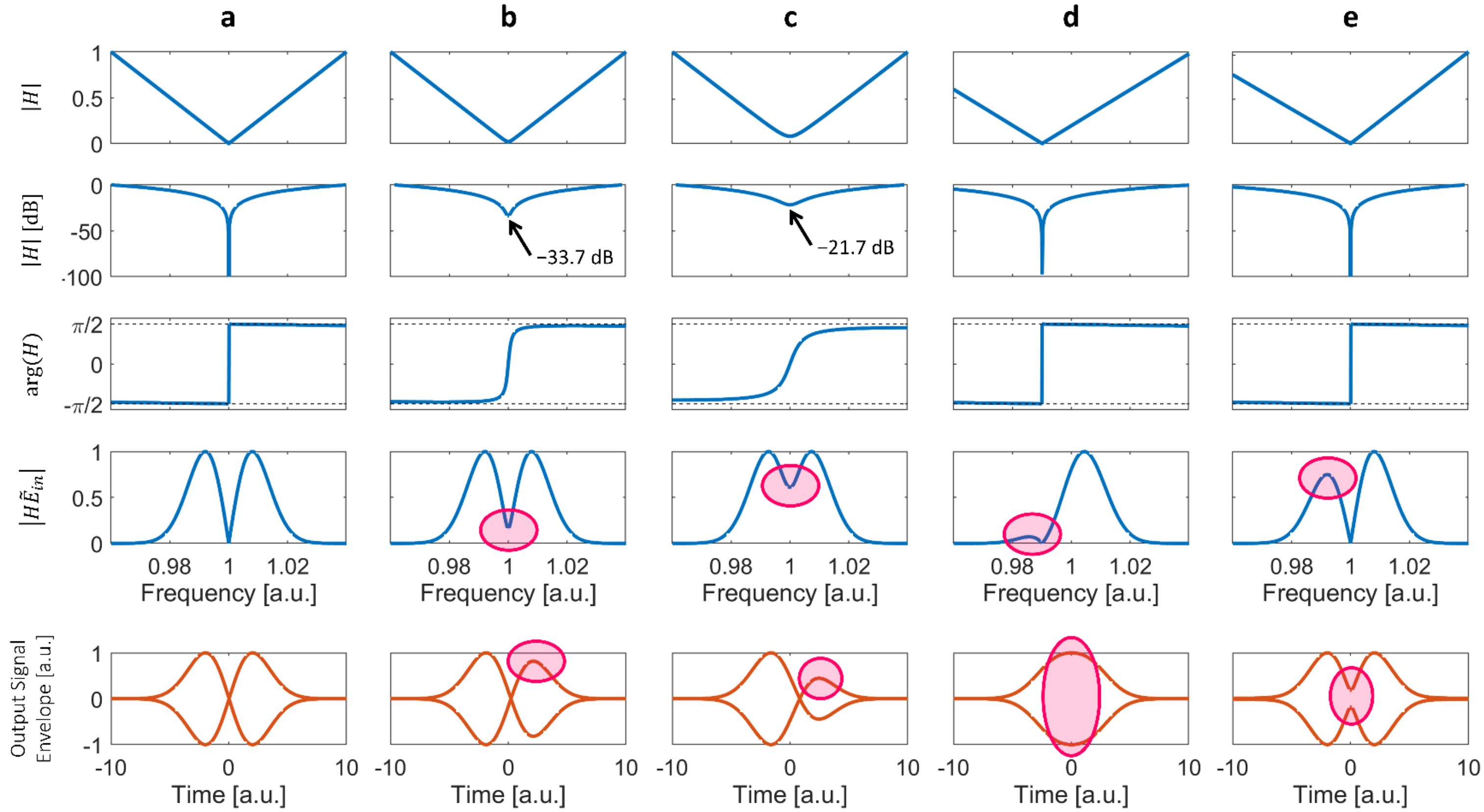

Supplementary Figure 1. Vulnerability of analog differentiators. For different transfer functions (**a-e**), the top row shows the magnitude of the transfer function on a linear scale, the second row shows the magnitude of the transfer function on a logarithmic scale, the third row shows the phase of the transfer function, the forth row shows the magnitude of the output signal's spectrum (for a Gaussian envelope as input signal), and the bottom row shows the envelope of the output signal (again for a Gaussian envelope as input signal). The different transfer functions correspond to: **a,** ideal; **b,** like ideal but with $A = 0.9975$; **c,** like ideal but with $A = 0.99$; **d,** like ideal but τ increased by a factor of 1.01; **e,** like ideal but amplitude of the transfer function for $\omega < \omega_0$ multiplied by a factor of 0.75.

not good enough to construct a faithful differentiator. In column **c**, we use $A = 0.99$ which makes the observations from **b** even more prominent.

In column **d**, we investigate the impact of deviating from the ideal case through a slight increase of the ideal value of τ by a factor of 1.01. This minimal change significantly shifts the frequency at which the zero lies on the real frequency axis to the left, such that the output signal has no resemblance with the analytically expected derivative of the Gaussian input envelope.

In column **e**, we investigate the role of asymmetry of the transfer function around ω_0. We alter the ideal transfer function by multiplying it with a factor of 0.75 for $\omega < \omega_0$. The output signal is again significantly distorted: it clearly does not have zero amplitude at its center in the time domain anymore, and its magnitude in the spectral representation is clearly asymmetric now.

These illustrative examples highlight the importance of *perfectly* placing a zero of the scattering matrix exactly on the real frequency axis and exactly at the desired frequency of operation.

Supplementary Note 4. Details on the Experimental Setup

In this supplementary note, we provide further details on the experimental setup, including characterizations of the chaotic cavity in terms of its reverberation time and of the programmable metasurface in terms of its operating bandwidth.

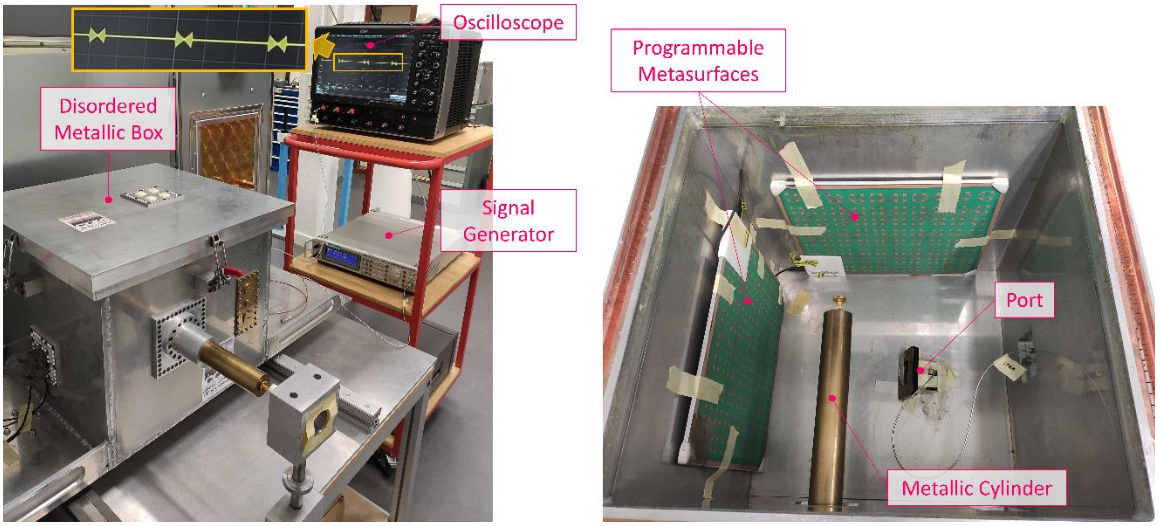

Supplementary Figure 2. Photographic image of the experimental setup. (right) Overview of experimental setup involving the disordered metallic box, the signal generator (Aeroflex IFR 3416, 250kHz-6GHz) and the oscilloscope (SDA 816Zi-B 16GHz Serial Data Analyzer, 80GS/s). In the pictured instance, the signal generator injects a waveform with quadratic envelope (signal power: 2dBm; carrier frequency: 5.1GHz) into the system, and the corresponding output signal is seen on the oscilloscope's display: a linear envelope. (left) Inside view of the metallic box with the top cover removed. Two programmable metasurfaces are placed on perpendicular walls, and a waveguide-to-coax adapter (RA13PBZ012-B-SMA-F) acts as port. A metallic cylinder breaks the symmetries of the box and ensures that the rays therein display chaotic behavior.

A photographic image of the setup corresponding to Figure 1 from the main text is shown in Supplementary Figure 2. The metallic box has dimensions of $0.385\mathrm{m} \times 0.422\mathrm{m} \times 0.405\mathrm{m}$ and hence a volume of $0.0658\mathrm{m}^3$. Chaotic behavior of the waves inside the metallic box is ensured via various mechanisms: on the one hand, the presence of metallic elements (notably the cylinder but also the port itself as well as irregularities on the wall) breaks the symmetries of the box; on the other hand, the use of metasurface configurations in which the meta-atoms are not all in the same state acts as an electronic equivalent of further geometric perturbations[9]. We determine the enclosure's quality factor as $Q = 410$

via the average decay rate of inverse Fourier transformed spectra measured for a series of random metasurface configurations. Based on Weyl's law, this means that at a given frequency within the considered interval $N \sim \frac{8\pi V}{c^3 Q} f_0^3 = \frac{8\pi}{Q} \frac{V}{\lambda_0^3} = 21$ modes overlap.

The programmable metasurface is an ultrathin array of meta-atoms whose electromagnetic scattering properties can be reconfigured electronically. The concept can be traced back to pioneering works in the early 2000s[10,11] and received renewed attention in 2014[12,13]. Since then, many designs for programmable metasurfaces have been proposed, and such devices are also known as "tunable impedance surface", "spatial microwave modulator" or "reconfigurable intelligent surface". Our present work could be implemented with any programmable metasurface design because it solely relies on the ability to somehow tune the complex scattering system's properties but not on the specific meta-atom properties. An ideal programmable metasurface for our purpose (i) interacts with as many rays as possible, meaning that each meta-atom has the largest possible scattering cross-section and the metasurface consists of as many meta-atoms as possible, (ii) the meta-atom programmability is as fine-grained as possible (but at least 1-bit), and (iii) insertion of the metasurface into the chaotic enclosure does not significantly alter the amount of absorption.

The prototype used in our experiments (purchased from Greenerwave) is based on the design introduced in Ref.[13] and has previously been used in Refs.[9,14–18]. In our setup seen in Figure 1 of the main text and Supplementary Figure 2, the programmable metasurfaces cover 16.2% of the cavity's wall surface. In the setup seen in Figure 4a of the main text, the programmable metasurface covers 8.1% and 7.2% of the wall surface area in the two cavities, respectively. Each meta-atom (see Supplementary Figure 3a) has two digitalized states, "0" and "1", and can be toggled between these two states by controlling the bias voltage of an integrated PIN diode. Specifically, each meta-atom consists of two resonators that hybridize, as detailed in Ref.[13], and via the bias voltage of a PIN diode the resonance frequency of one of the two resonators can be altered. Thereby, the phase change of the reflected wave can be tuned by roughly π. The meta-atoms in Ref.[13] acted on a single field polarization, whereas the meta-atoms of the prototype that we utilize can be thought of as the fusion of two such meta-atoms, one rotated by 90°, each acting on one polarization of

the electromagnetic field.

We begin by characterizing the utilized programmable metasurface in the conventional way, namely to consider the phase shift of the reflected wave between the two possible states for normally incident waves. The metasurface consists of 76 of the above-described meta-atoms. To that end, we utilize the horn-antenna setup shown in Supplementary Figure 3a and synchronize the states of all meta-atoms for this measurement. We measure the return loss of the horn antenna when the meta-atoms are all simultaneously in their two possible states: “0” or “1”. The magnitude of the return loss in the two cases, as well as the phase difference of the return loss between the two cases, are plotted in Supplementary Figure 3b,c. The return loss magnitudes are of course modulated by the horn antenna’s transfer function and thus not a quantification of the energy that is absorbed by the metasurface, but it is apparent that there is no significant difference in terms of reflected field magnitude between the two states. The phase difference reaches the ideal value of π in the vicinity of 5.15 GHz. In other words, at this frequency, every meta-atom can be configured to mimic Dirichlet or Neuman boundary conditions. Similar results were measured for the other field polarization.

The above “conventional” characterization is a useful first indication of the metasurface’s properties but of course it contains no information about the dependence of these properties on the angle of incidence or on the coupling between different meta-atoms. Especially in a rich scattering setting such as the one we consider, in which certainly waves from all possible angles are incident on the metasurface, it is more meaningful to characterize the metasurface *in situ*[19,20]. To that end, we place the two programmable metasurfaces at their intended locations, as seen in Supplementary Figure 3d, and measure the scattering parameter of interest (here $S_{11}(f)$) for a series of 500 random metasurface configurations. By evaluating the standard deviation across these measurements, we obtain a useful metric to assess the extent to which the programmable metasurface impacts the considered scattering parameter in the considered setup. Of course, this metric is still somewhat influenced by the specific system, so we also plot a smoothed version of the curve, obtained with a sliding average filter. Overall, it can be seen that the programmable metasurface most efficiently modulates the field within a 400 MHz bandwidth centered roughly on 5.15 GHz.

For the experiment underlying Figure 4 in the main text, we use an additional second metallic box of dimensions $0.5\text{m}\times 0.5\text{m}\times 0.3\text{m}$ and hence of volume of 0.075m^3. Chaotic behavior of the waves inside the metallic box is ensured via various mechanisms: on the one hand, the presence of metallic elements (notably two metallic hemispheres but also the port itself as well as irregularities on the wall) breaks the symmetries of the box; on the other hand, the use of metasurface configurations in which the meta-atoms are not all in their perfect electric conductor (PEC) equivalent state acts as an electronic equivalent of further geometric perturbations[9]. We determine the enclosure's quality factor as $Q = 446$ via the average decay rate of inverse Fourier transformed spectra measured for a series of random metasurface configurations. Based on Weyl's law, this means that $N \sim \frac{8\pi V}{c^3 Q} f_0^3 = \frac{8\pi}{Q}\frac{V}{\lambda_0^3} = 22$ modes overlap at a given frequency within the considered interval.

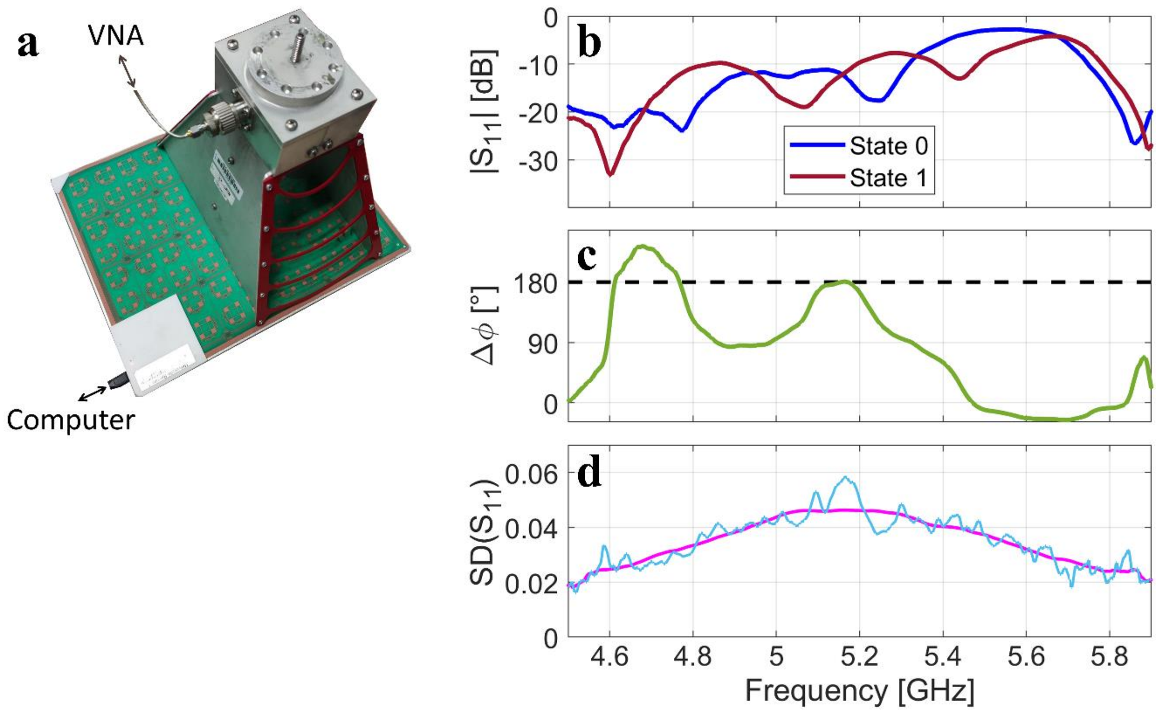

Supplementary Figure 3. Characterization of the programmable metasurface. a, Setup to characterize the response under normal incidence. **b,** Magnitude of the return loss measured with the setup from **a** when all meta-atoms are simultaneously either in state "0" or state "1". **c,** Phase difference between the return losses measured with the setup in **a** in the two possible states. **d,** *In situ* characterization of the metasurface via the standard deviation of the reflection spectrum measured inside the disordered metallic box for 500 random metasurface configurations. A smoothed version of the curve is also shown.

Supplementary Note 5. Details on the Experimental Procedure

In this supplementary note, we discuss procedural details of our experiments, including the identification of suitable metasurface configurations, the direct observation of temporal differentiation of various waveforms, as well as details regarding the modified setups underlying Figures 3 and 4 of the main text.

In order to separate injected and reflected waveform, port 2 of a circulator (PE83CR006) is connected to the coax-to-waveguide adapter inside the metallic disordered box, as seen in Supplementary Figure 4.

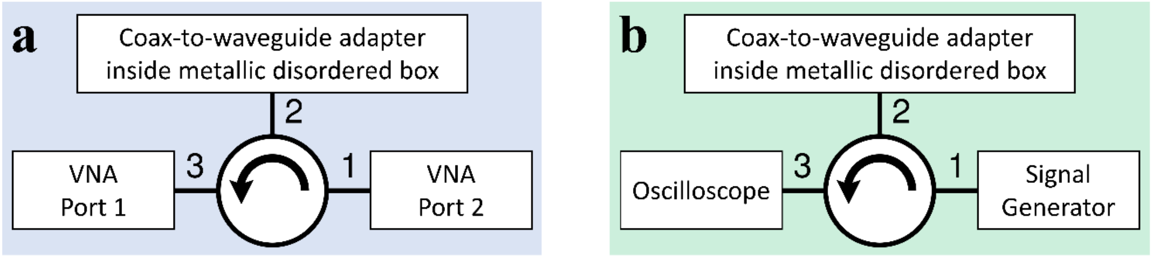

Supplementary Figure 4. Use of a circulator to separate injected and reflected signals. a, Port connections involving a VNA used to optimize the metasurface configuration. **b,** Port connections involving a signal generator and oscilloscope used to perform temporal differentiation.

Identifying a metasurface configuration that yields a zero at a desired frequency is not a trivial task because no analytical or learned forward model is available that would allow us to predict the scattering response which corresponds to a given metasurface configuration. Given that the amount of degrees of freedom in the present work is one order of magnitude larger than in Ref.[21], measuring the scattering response for all possible metasurface configurations is intractable. Instead, we use an iterative experimental trial-and-error optimization method. To that end, we connect a vector network analyzer (Agilent Technologies PNA-L Network Analyzer N5230C, 300kHz-20GHz) to ports 1 and 3 of the circulator, as shown in Supplementary Figure 4a. We operate with an emitted power of 0 dBm and an IF bandwidth of 10 kHz. The transmission from port 1 to port 3 of the circulator is the reflection off the port inside the disordered metallic box. First, we measure the scattering response for 100 random metasurface configurations. We pick the one closest to our objective as starting point. Then, we flip the state of one meta-atom at a time; we keep

the change if the resulting scattering response is closer to our objective. We observe that typically after a maximum of roughly 700 iterations no further improvement is observed. Like most inverse design methods, ours does not guarantee the identification of the globally optimal metasurface configuration. However, we observe that different optimization runs yield outcomes of comparable quality. In the future, we are confident that learned forward models based on ANNs can be implemented, such that the need for this iterative experimental optimization can be circumvented. Ref.[22] already employs a learned forward model to predict the scattering response of a programmable metasurface, albeit in quasi free space rather than inside a complex scattering enclosure. Such ANN-based approaches will greatly benefit from next-generation meta-atoms with fine-grained programmability ($>$ 1-bit) because they become compatible with continuous gradient-descent optimization protocols. Note that in certain settings without environmental perturbations during runtime, the identification of suitable metasurface configurations can be completed offline during a calibration phase and presents no burden during runtime.

To directly observe the wave-based analog computation of temporal derivatives, we generate various waveforms with a signal generator and observe the reflected signals on an oscilloscope, with connections as shown in Supplementary Figure 4b. Specifically, for the main experiments we use a signal generator (Aeroflex IFR 3416, 250kHz-6GHz) to generate a signal $e(t)e^{i\omega_0 t}$. An arbitrary signal envelope $e(t)$ is defined and sampled at 33 MHz, and an arbitrary value of ω_0 can be chosen within the considered 5-GHz-band. We emit the signals at 2 dBm. The emitted signal envelopes (GAUSSIAN, QUADPOLY, SKYLINE) are displayed in Figures 2c,f,i in the main text. The signal envelopes are repeated in intervals of 3 μs. The oscilloscope (SDA 816Zi-B 16GHz Serial Data Analyzer 80GS/s) measures a 10 μs interval of the reflected signal with a sampling rate of 40 GS/s.

In order to inject the sum of two signals $e_A(t)e^{i\omega_A t} + e_B(t)e^{i\omega_B t}$ for the experiments underlying Figure 3 of the main text, we use a second signal generator (Agilent MXG Analog N5183A, 100kHz-20GHz). Signal generators A and B generate $e_A(t)e^{i\omega_A t}$ and $e_B(t)e^{i\omega_B t}$, respectively, and the two generated signals are then summed using a simple "T" connector before being injected into the port. The second signal generator generates a square wave with period 2.5 μs at -13 dBm and an arbitrary value can be chosen for ω_0. The reason for emitting at such low power is that the peaks of the square wave's derivatives

are very large and we intend to measure them with the same dynamic range as the derivatives of the other signal envelopes (GAUSSIAN, QUADPOLY, SKYLINE). The two signals from generators A and B are not synchronized in any manner and have different repetition intervals.

To suppress measurement noise, all measured data is digitally filtered to impose a pass-band of $f_0 \pm 0.02\ \mathrm{GHz}$, where f_0 is the carrier frequency. The reflected signal's amplitude is intrinsically low for a differentiator due to the zero at the carrier frequency in its transfer function. Hence, the output signal is particularly weak in the case of a second-order differentiator as considered in Figure 4 of the main text. Therefore, the output signal envelopes displayed in Figure 4 of the main text are averaged over 20 acquisitions in order to limit the corruption through measurement noise.

Supplementary Note 6. Experimentally Measured Notch Depths

In this supplementary note, we provide further details on the experimentally measured notch depths for the data displayed in Figure 2a of the main text. To that end, we reproduce Figure 2a,b from the main text in Supplementary Figure 5 and add a plot of the transfer function magnitude on a logarithmic scale. Moreover, we summarize the notch depth for each considered central frequency in a table below.

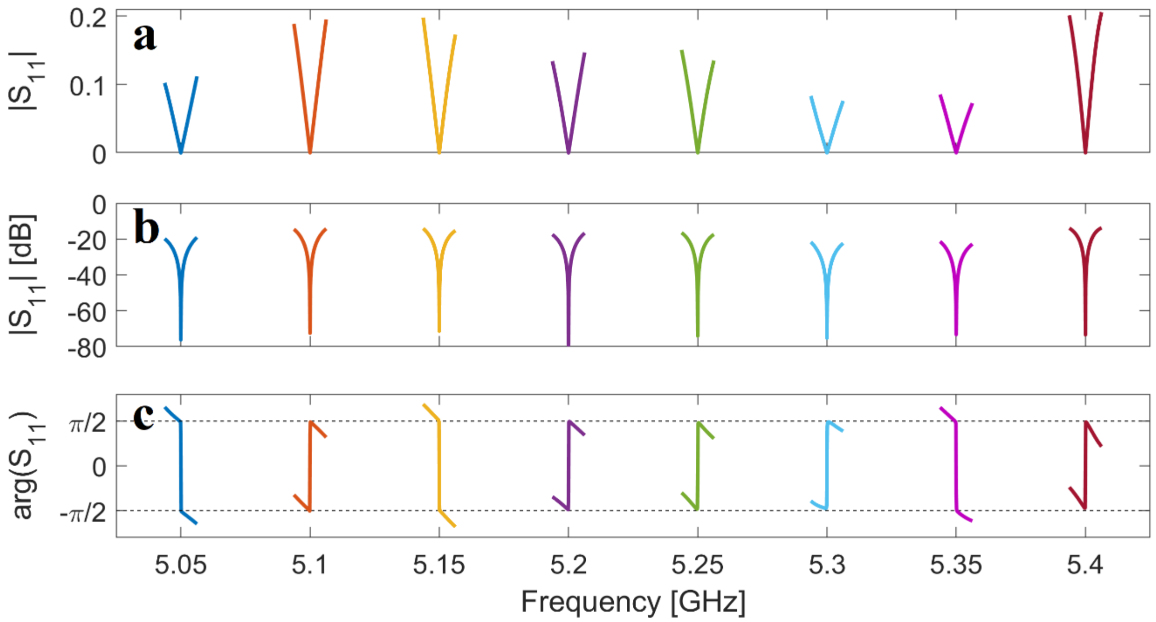

Supplementary Figure 5. Experimentally measured notch depths. Measured transfer functions in terms of magnitude on a linear scale (**a**), magnitude on a logarithmic scale (**b**), and phase (**c**). Subfigures **a** and **c** are reproduced from Figure 2a,b in the main text.

**Central Frequency [GHz]**	**Notch Depth [dB]**
5.05	-76.3
5.10	-72.5
5.15	-71.4
5.20	-82.0
5.25	-74.1
5.30	-75.3
5.35	-73.3
5.40	-73.5

Supplementary Note 7. Error Performance Evaluation: Bandwidth and Dip Symmetry

In this supplementary note, we analyze the computation error of our meta-programmable differentiator based on a measured system transfer function. We explore the error dependence on the input signal's bandwidth and discuss the role of the symmetry of the reflection dip.

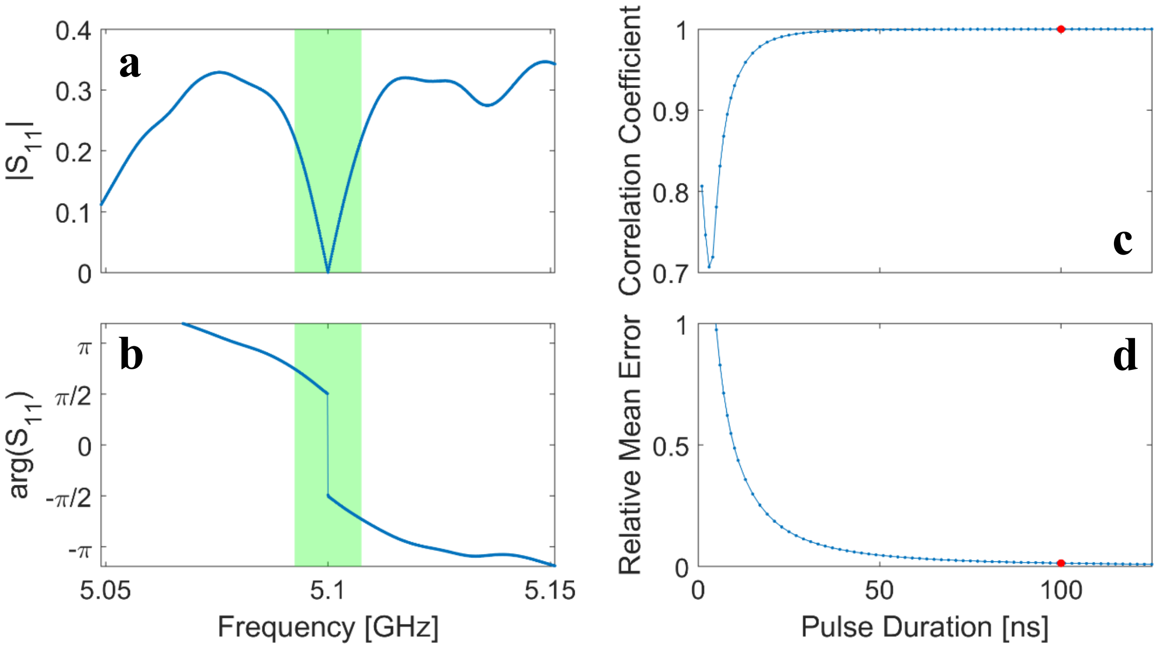

Supplementary Figure 6. Evaluation of computation error as a function of input signal bandwidth. a,b, Magnitude (**a**) and phase (**b**) of the measured transfer function with a zero at 5.1 GHz that is used for the error evaluation. The shaded area indicates the bandwidth of 15 MHz within which this transfer function is a good approximation to that of an ideal differentiator. **c,d,** Computational error as a function of input signal duration in terms of two metrics: correlation coefficient (**c**) and relative mean error (**d**). To compute these metrics, the output signal for a given input pulse duration is calculated, normalized and compared to the analytically expected output signal. The red dot indicates the duration of the Gaussian pulse used in the main text (Figure 2c).

The ideal differentiator's transfer function is $H(\omega) = i(\omega - \omega_0)$ – see Supplementary Note 2; its magnitude is hence linear and symmetric with respect to ω_0. Any physical implementation of this transfer function is only an approximation within some bandwidth around ω_0 and hence intrinsically bandwidth-limited. Most wave-based

differentiators that have been proposed so far (see references in the main text's introduction) are based on regularly shaped devices and their scattering matrix often only has a single zero within the considered frequency range. In contrast, our overmoded random scattering system has typically many other poles and zeros (with non-zero imaginary component) in the vicinity of the zero that we tune to a real frequency. These are, of course, in principle not distributed symmetrically around the targeted frequency and can hence introduce strong asymmetries outside the direct vicinity of the real-valued zero. Therefore, our optimization protocol not only minimizes $|H(\omega_0)|$ but also pays attention to the symmetry of $|H(\omega)|$ in the vicinity of ω_0. Indeed, different locally optimal metasurface configurations that minimize $|H(\omega_0)|$ have been found to sometimes vary drastically in terms of symmetry and thus useful bandwidth. The stronger the symmetry is, the larger is the maximum input signal bandwidth for which an acceptably low computation error can be guaranteed.

Considering the case of an ideal Gaussian pulse as input (similar to Figure 2c in the main text), we calculate the (normalized) output signal envelope and compare it to the analytically expected output signal envelope for an ideal differentiator using two metrics: the relative mean difference between the two, as well as the correlation coefficient of the two. This calculation is performed for various bandwidths of the input pulse. The results in Supplementary Figure 6 show that up to an input signal bandwidth of roughly 15 MHz our system's performance is very close to that of an ideal differentiator.

In the table below, we compare the fractional bandwidth achieved by various wave-based temporal differentiators reported in the literature. Refs.[23–26] are static (not programmable) wave-based photonic differentiators, while Ref.[8] is a reconfigurable integrated interferometric photonic differentiator. None of these devices experiences symmetry issues regarding the transfer function magnitude (as discussed above) but implementation-specific effects eventually cause deviations from the ideal linear shape; for instance, in Ref.[23] due to a slight non-linear dispersion slope of the core and cladding modes. In terms of fractional bandwidth, a quantity that is independent of the central operating frequency and hence most meaningful from a general wave engineering perspective, our device achieves a performance of the same order of magnitude as Ref.[26] and only Ref.[23] performs significantly better. Refs.[23,26] are both static and the only report on a somewhat tunable differentiator in Ref.[8] has a fractional bandwidth which is an order of magnitude

lower than the one reported in this present work. Interestingly, Refs.[8,25] observed that the computational error deteriorates if the input bandwidth gets too large *or too small* – unlike our work in which only too large signal bandwidths appear to deteriorate the computational precision. Finally, we note that our meta-programmable analog differentiator's operating bandwidth of 15 MHz is of the same order of magnitude as the channel width of typical WLAN wireless communication channels operating at the same central frequency – paving the path to direct applications of our technique in this area.

Reference	Operation Principle	Central Frequency f_0	Frequency Bandwidth Δf	Fractional Bandwidth $\Delta f/f_0$
Ref.[23]	Long-period fiber grating.	195 THz	2.3 THz	1.5×10^{-2}
Ref.[24]	Microring resonator.	193 THz	42 GHz	2×10^{-4}
Ref.[25]	Fiber Bragg grating.	193.5 THz	25 GHz	1×10^{-4}
Ref.[26]	Directional coupler.	193.5 THz	1.25 THz	6.5×10^{-3}
Ref.[8]	Tunable interferometer.	192 THz	55 GHz	3×10^{-4}
This work.	Tuned overmoded chaotic cavity.	5.1 GHz	15 MHz	3×10^{-3}

Supplementary Note 8. Operation in Transmission Mode

In the main text, we presented results obtained in reflection mode. In this supplementary note, we present complementary results obtained in transmission mode for a first-order temporal differentiator. In reflection mode, a single port is used but a circulator is necessary to separate incident and reflected signal (see Supplementary Figure 4); in transmission mode, two ports are required but there is no need for a circulator.

The main inconvenience of operating in transmission mode is the significantly weaker magnitude of the transfer function in comparison to operating in reflection mode; in our experiments, the transfer function magnitude, averaged over random metasurface configurations and all frequency points, is $17.74\ \mathrm{dB}$ lower if operating in transmission: $\langle|S_{11}|\rangle = -15.22\ \mathrm{dB}$ in contrast to $\langle|S_{21}|\rangle = -27.96\ \mathrm{dB}$. This difference in transfer function magnitude results in a drastically higher sensitivity to noise during the optimization of the metasurface configuration, as well as less energy-efficient analog wave processing, if operation in transmission mode is chosen.

Nonetheless, V-shaped transmission zeros can be imposed on demand analogously to the V-shaped reflection zeros discussed in the main text, and the resulting transfer function is that of a first-order differentiator. Refs.[19,27] previously minimized the transmission between two ports in comparable metasurface-tunable complex scattering settings to create "cold spots", but these works did not implement true transmission zeros. An example of a transmission zero that we implemented in our transmission-mode setup is shown in Supplementary Figure 7 below. In comparison to the reflection-mode setup detailed in Figure 1 in the main text, we removed the circulator and instead added a second port inside the chaotic cavity. The second port is identical to the first port and placed at least half a wavelength away from the first port, in perpendicular orientation to the first port. The transfer function's magnitude has the desired linear behavior around its minimum at $-79.5\ \mathrm{dB}$ and the phase displays the required π phase jump across the central frequency. Again, a global phase drift is seen, which does not impact the differentiator functionality (see Supplementary Note 2). Upon injection of a Gaussian pulse (same parameters as in the main text), the corresponding output measurement yields the curve displayed in Supplementary Figure 7d. The principal features of the analytically expected output signal envelopes are observed (two symmetric pulses and a zero between them), but the quality of the differentiation operation is clearly worse than that reported in the main text in

reflection mode. The very low signal strength in comparison to the noise floor is apparent; neater results could be obtained by averaging over multiple acquisitions.

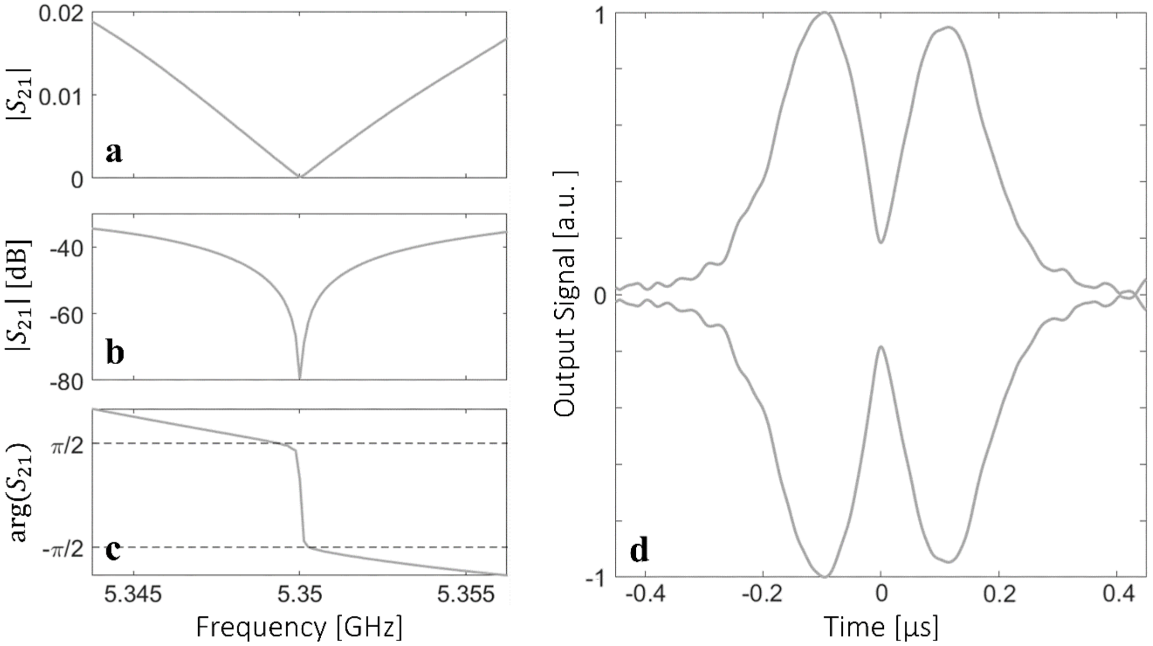

Supplementary Figure 7. Example of an analog differentiator operating in transmission mode at 5.35 GHz. a,b,c, The left column displays the transfer function, here $S_{21}(f)$, in terms of its absolute value on a linear (**a**) and logarithmic (**b**) scale, as well as the associated phase (**c**). **d,** Normalized envelope of the measured output signal (upon injection of a Gaussian pulse).

Supplementary Note 9. Further Examples of Parallel Computing

In the main text, we provided in Figure 3 several examples of computing two derivatives simultaneously by imposing simultaneously two zeros on the transfer function at distinct frequencies ω_A and ω_B. We reported direct evidence of parallel computing by injecting the sum of two independent envelopes modulated onto carriers ω_A and ω_B, respectively. In this supplementary note, we provide further examples of measured optimized transfer functions for parallel differentiation, for up to four independent data streams. Given that only two signal generators were at our disposal, we could not directly test these further examples of parallel computing which would require the simultaneous generation of three or four independent waveforms.

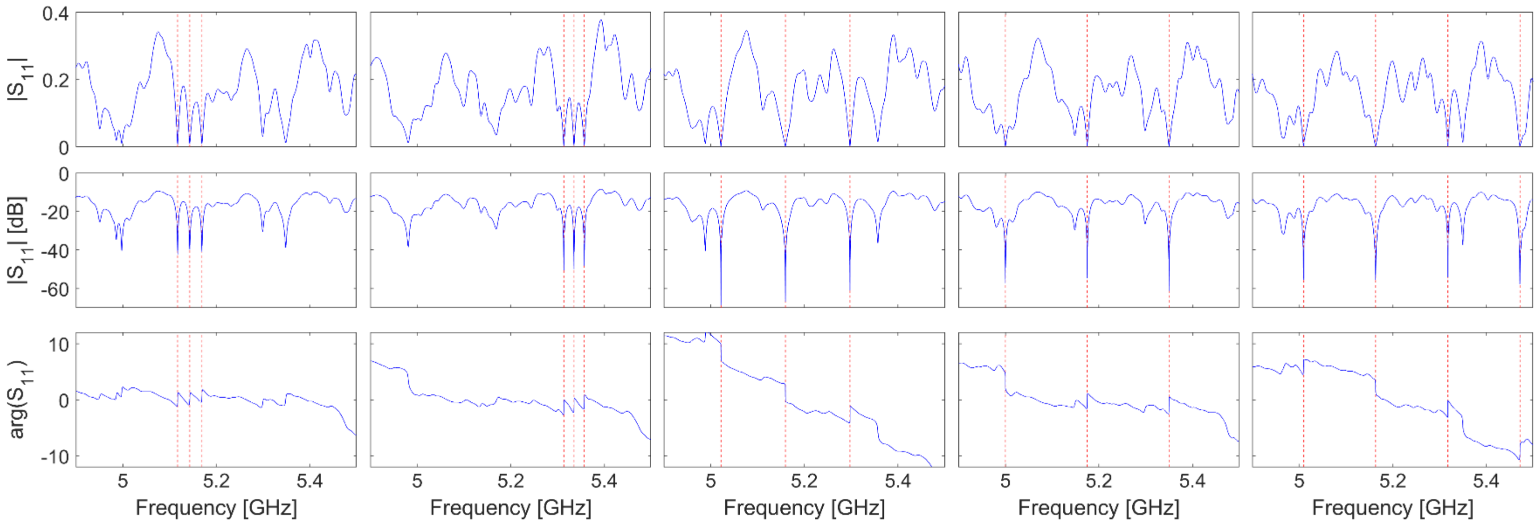

Supplementary Figure 8. Various examples of optimized transfer functions with multiple zeros for parallelized wave-based differentiation. For each example, the transfer function's amplitude is shown on a linear scale (top row) and on a logarithmic scale (middle row), as well as the corresponding phase (bottom row). Four examples with three scattering zeros and one example with four scattering zeros are shown.

Five examples of experimentally optimized and measured transfer functions with three or four real-valued scattering matrix zeros in the considered 5-GHz-band are displayed in Supplementary Figure 8. The regular spacing of these zeros was chosen on purpose. It is apparent that closely spaced zeros are more difficult to impose (the first two examples in Supplementary Figure 8). However, in all shown cases the reflection dips are sufficiently deep to serve for the desired differentiation functionality. An example with four zeros is

also shown in Supplementary Figure 8 (last example). In principle, such multi-objective optimizations are more demanding than imposing a single zero. To ease the optimization burden, depending on the requirements of the specific intended application it may be possible to relax constraints (for instance, to only fix the zeros' spacing but not their exact positions; to only fix the number of desired zeros; etc.). Alternatively, more degrees of freedom by using a larger programmable metasurface can improve the ability to satisfy all constraints.

Finally, we point out that parallel computing as reported in our work based on an overmoded chaotic cavity with many tunable degrees of freedom is not possible with the same flexibility if a tunable interferometer is used instead as physical device, as in Ref.[8]. Indeed, in the latter case, the spacing of carrier frequencies must be equal to the free spectral range and cannot be imposed at will – see, for instance, Figure 3b in Ref.[8].

Supplementary Note 10. Generalizations

In the main text, we presented meta-programmable analog differentiation of temporally encoded signals through "over the air" wave propagation in a programmable complex scattering enclosure in the microwave domain. In this supplementary note, we provide details on how our proposed concept can be generalized to (i) *spatially* encoded information, (ii) programmable overmoded random scattering system based on *guided* waves, and (iii) *acoustic or optical* scattering.

(i) Spatially Encoded Information

To obtain the hallmark V-shaped transfer function in our work, as seen for instance in Figure 2, we impose a zero for the chosen set of parameters at which we desire critical coupling (in particular, ω_0); then, we trace the transfer function upon continuous detuning of the frequency, yielding the V-shape in the vicinity of the critical coupling condition. Thus, information that is temporally encoded into the input signal will be subject to the differentiation transfer function.

However, the same V-shaped spectrum is also obtained in the vicinity of the critical coupling condition by detuning any other parameter. Therefore, if the input signal is not incident through a mono-modal coaxial waveguide but carried by a wave propagating in free space that impinges at some angle θ on a perforated wall through which it couples to our programmable chaotic cavity, as sketched in Supplementary Figure 9, we can tune the system once again to critical coupling for a chosen set of parameters (in particular, θ_0); then, we can trace the transfer function upon continuously detuning the angle of incidence, yielding once again the V-shape in the vicinity of the critical coupling condition. This time, however, the horizontal axis will be the angle of incidence as opposed

to the frequency. Thus, information that is spatially encoded into the input signal (over a range of incidence angles) will be subject to the differentiation transfer function.

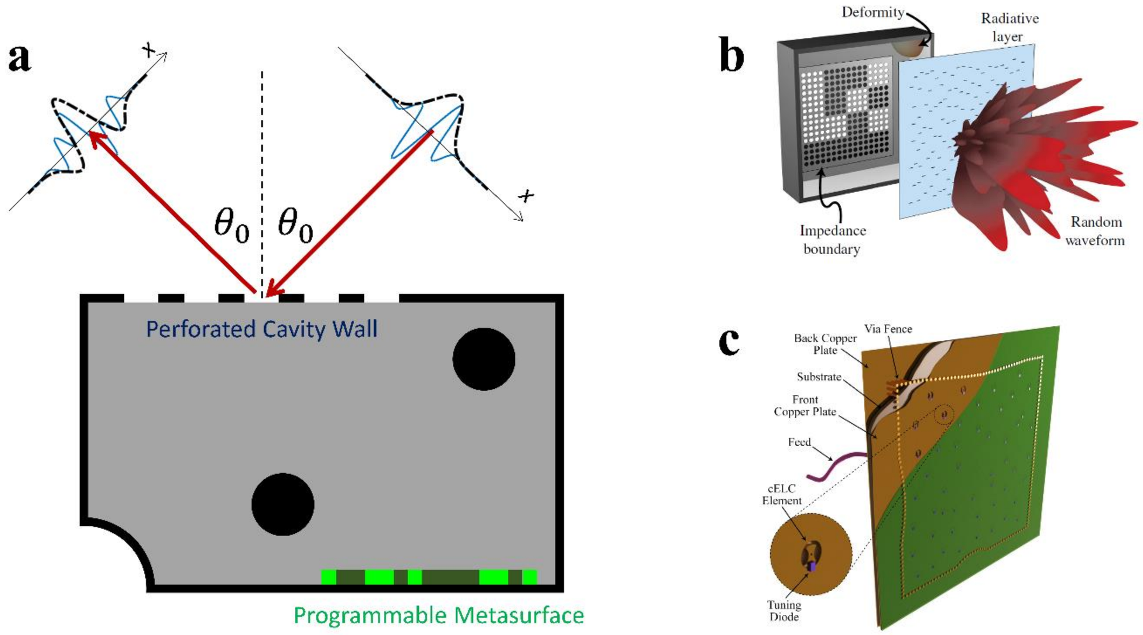

Supplementary Figure 9. Metaprogrammable *spatial* analog differentiator. a, Concept. **b,** Implementation in a 3D chaotic cavity from Ref.[28]. **c,** Implementation in a flat quasi-2D chaotic cavity from Ref.[29].

Therefore, our technique can straightforwardly be extended to meta-programmable spatial (as opposed to temporal) analog differentiation, and various technique to perforate a wall of 3D or quasi-2D chaotic cavities are well-known from setups that are routinely used for computational meta-imaging, e.g., in Refs.[28,29]. The optimization to tune a zero of the scattering matrix to the real frequency axis for the desired set of parameters (central frequency, angle of incidence) would be exactly the same as that used in the work we presented.

However, the differentiation of spatially as opposed to temporally encoded information appears more relevant to optical signal processing than to the

microwave domain which is targeted in our work. In the microwave domain, information is temporally encoded into many signals of interest, for instance, in wireless communication or radar.

(ii) Programmable Overmoded Random Scattering System Based on Guided Waves

At the core of our technique lies the idea to purposefully perturb an overmoded random scattering system with many degrees of freedom, because such a system presents a high density of zeros that are statistically uniformly distributed along the real frequency axis. Therefore, our technique finds it easy to tune one or multiple of these zeros onto the real frequency axis at the desired position(s). Our experimental demonstration relies on a 3D metallic complex scattering enclosure because of its high practical relevance: The elegance of our experiments is that any metallic enclosure (toolbox, microwave oven, etc.) can be taken and only an ultrathin programmable metasurfaces needs to be attached to the walls. In the context of upcoming 6G wireless communication, many rooms which are also scattering enclosures at $< 6\,\mathrm{GHz}$ will naturally be equipped with "reconfigurable intelligent surfaces" such that the entire hardware needed to implement our concept "*over the air*" is already there.

Nonetheless, our concept can also be implemented in programmable overmoded random scattering systems based on *guided* waves, such as a network of coupled transmission lines with complex connectivity (also known as graph)[30,31]. The programmability could then originate, for instance, from a series of phase shifters integrated into the connections between nodes of the graphs. Indeed, recently CPA has been experimentally observed also on chaotic graphs in Ref.[32] (albeit not "on-demand" as in our work in order to impose CPA

at any desired real frequency). In contrast to our "over-the-air" experimental implementation, a guided approach would require one to carry around a network of transmission lines unless there was a safe method to leverage, for instance, the wiring inside a building that exists for electricity supply.

(iii) Toward Implementations in Acoustic and Optical Scattering

The experiments reported in our manuscript were conducted in the microwave domain not merely because this is a convenient regime for proof-of-principle experiments but because it is of direct technological relevance in sight of many examples of microwave signals carrying temporally encoded information, such as in wireless communication or radar. Nonetheless, the fundamental principles underlying our technique are generic to any wave scattering system and can hence be extended to different parts of the electromagnetic spectrum as well as to acoustic scattering, provided a suitable mechanism to implement individually programmable scatterers can be identified. In this section, we provide a succinct survey of recent works from the literature that implemented techniques which may be leveraged to implement our proposal, for instance, in acoustic or optical scattering.

To begin with, we note that programmable meta-atoms very similar to the microwave prototype we used can be implemented at much higher electromagnetic frequencies in the THz and even infrared regimes using several well-established switchable diodes. For example, Schottky diodes in the THz regime (see Ref.[33] and the website of Teratech Components Ltd: http://www.teratechcomponents.com/) and thermal VO_2 diodes in the infrared regime (see Refs.[34,35]) can be used. Detailed design proposals for programmable meta-atoms based on these switchable diodes for operation around 350 GHz and 150 THz, respectively, can be found in the electronic supplementary material of Ref.[36].

In the optical regime, programmable scatterers have been demonstrated based on electro-optical modulation[37], phase-change materials[38–40], applied-voltage-sensitive graphene[41–43], all-optical modification of the spatial refractive index profile[44], acousto-optic modulation[45], spatial light modulators (SLM, if the scattering system is defined as the ensemble of complex medium and SLM)[46], as well as mechanical actuation[47,48] and computer-controlled mechanical perturbations[49].

In the acoustic regime, programmable scatterers have been demonstrated based on magnetic-field controlled elastomers[50], tunable membranes[51–54], geometrically-tunable resonators[55–59], as well as electrolysis-controlled microbubble arrays for ultrasound[60].

The existence of such a variety of experimental reports on tunable scattering in optics and acoustics suggests that our concept can be implemented with relative ease for optical or acoustic scattering, too.

Supplementary References

1. Chong, Y. D., Ge, L., Cao, H. & Stone, A. D. Coherent Perfect Absorbers: Time-Reversed Lasers. *Phys. Rev. Lett.* **105**, 053901 (2010).
2. Dhia, A.-S. B.-B., Chesnel, L. & Pagneux, V. Trapped modes and reflectionless modes as eigenfunctions of the same spectral problem. *Proc. R. Soc. A.* **474**, 20180050 (2018).
3. Sweeney, W. R., Hsu, C. W. & Stone, A. D. Theory of reflectionless scattering modes. *Phys. Rev. A* **102**, 063511 (2020).
4. Grimm, P., Razinskas, G., Huang, J.-S. & Hecht, B. Driving plasmonic nanoantennas at perfect impedance matching using generalized coherent perfect absorption. *Nanophotonics* **10**, 1879–1887 (2021).
5. Pichler, K. *et al.* Random anti-lasing through coherent perfect absorption in a disordered medium. *Nature* **567**, 351–355 (2019).
6. Rotter, S. & Gigan, S. Light fields in complex media: Mesoscopic scattering meets wave control. *Rev. Mod. Phys.* **89**, 015005 (2017).
7. Park, Y., Azaña, J. & Slavík, R. Ultrafast all-optical first- and higher-order differentiators based on interferometers. *Opt. Lett.* **32**, 710 (2007).
8. Liu, W. *et al.* A fully reconfigurable photonic integrated signal processor. *Nat. Photonics* **10**, 190–195 (2016).
9. Gros, J.-B., del Hougne, P. & Lerosey, G. Tuning a regular cavity to wave chaos with metasurface-reconfigurable walls. *Phys. Rev. A* **101**, 061801 (2020).
10. Sievenpiper, D. F., Schaffner, J. H., Song, H. J., Loo, R. Y. & Tangonan, G. Two-dimensional beam steering using an electrically tunable impedance surface. *IEEE Trans. Antennas Propag.* **51**, 2713–2722 (2003).
11. Holloway, C. L., Mohamed, M. A., Kuester, E. F. & Dienstfrey, A. Reflection and Transmission Properties of a Metafilm: With an Application to a Controllable Surface Composed of Resonant Particles. *IEEE Trans. Electromagn. Compat.* **47**, 853–865 (2005).
12. Cui, T. J., Qi, M. Q., Wan, X., Zhao, J. & Cheng, Q. Coding metamaterials, digital metamaterials and programmable metamaterials. *Light Sci. Appl.* **3**, e218–e218 (2014).
13. Kaina, N., Dupré, M., Fink, M. & Lerosey, G. Hybridized resonances to design tunable binary phase metasurface unit cells. *Opt. Express* **22**, 18881 (2014).
14. del Hougne, P., Davy, M. & Kuhl, U. Optimal Multiplexing of Spatially Encoded Information across Custom-Tailored Configurations of a Metasurface-Tunable Chaotic Cavity. *Phys. Rev. Applied* **13**, 041004 (2020).
15. del Hougne, P., Savin, D. V., Legrand, O. & Kuhl, U. Implementing nonuniversal features with a random matrix theory approach: Application to space-to-configuration multiplexing. *Phys. Rev. E* **102**, 010201 (2020).
16. del Hougne, P., Yeo, K. B., Besnier, P. & Davy, M. Coherent Wave Control in Complex Media with Arbitrary Wavefronts. *Phys. Rev. Lett.* **126**, 193903 (2021).
17. del Hougne, P., Yeo, K. B., Besnier, P. & Davy, M. On-Demand Coherent Perfect Absorption in Complex Scattering Systems: Time Delay Divergence and Enhanced Sensitivity to Perturbations. *Laser Photonics Rev.* **15**, 2000471 (2021).

18. del Hougne, P. *et al.* Diffuse field cross-correlation in a programmable-metasurface-stirred reverberation chamber. *Appl. Phys. Lett.* **118**, 104101 (2021).
19. Kaina, N., Dupré, M., Lerosey, G. & Fink, M. Shaping complex microwave fields in reverberating media with binary tunable metasurfaces. *Sci. Rep.* **4**, 6693 (2015).
20. Alexandropoulos, G. C., Shlezinger, N. & del Hougne, P. Reconfigurable Intelligent Surfaces for Rich Scattering Wireless Communications: Recent Experiments, Challenges, and Opportunities. *IEEE Commun. Mag.* **59**, 28–34 (2021).
21. Imani, M. F., Smith, D. R. & del Hougne, P. Perfect Absorption in a Disordered Medium with Programmable Meta-Atom Inclusions. *Adv. Funct. Mater.* **30**, 2005310 (2020).
22. Li, H.-Y. *et al.* Intelligent Electromagnetic Sensing with Learnable Data Acquisition and Processing. *Patterns* **1**, 100006 (2020).
23. Slavík, R., Park, Y., Kulishov, M., Morandotti, R. & Azaña, J. Ultrafast all-optical differentiators. *Opt. Express* **14**, 10699 (2006).
24. Liu, F. *et al.* Compact optical temporal differentiator based on silicon microring resonator. *Opt. Express* **16**, 15880 (2008).
25. Li, M., Janner, D., Yao, J. & Pruneri, V. Arbitrary-order all-fiber temporal differentiator based on a fiber Bragg grating: design and experimental demonstration. *Opt. Express* **17**, 19798 (2009).
26. Huang, T. L., Zheng, A. L., Dong, J. J., Gao, D. S. & Zhang, X. L. Terahertz-bandwidth photonic temporal differentiator based on a silicon-on-isolator directional coupler. *Opt. Lett.* **40**, 5614 (2015).
27. Frazier, B. W., Antonsen, T. M., Anlage, S. M. & Ott, E. Wavefront shaping with a tunable metasurface: Creating cold spots and coherent perfect absorption at arbitrary frequencies. *Phys. Rev. Research* **2**, 043422 (2020).
28. Sleasman, T., Imani, M. F., Gollub, J. N. & Smith, D. R. Microwave Imaging Using a Disordered Cavity with a Dynamically Tunable Impedance Surface. *Phys. Rev. Applied* **6**, 054019 (2016).
29. Sleasman, T. *et al.* Implementation and characterization of a two-dimensional printed circuit dynamic metasurface aperture for computational microwave imaging. *IEEE Trans. Antennas Propag.* **69**, 2151 (2020).
30. Kottos, T. & Smilansky, U. Chaotic Scattering on Graphs. *Phys. Rev. Lett.* **85**, 968–971 (2000).
31. Pluhař, Z. & Weidenmüller, H. A. Universal Chaotic Scattering on Quantum Graphs. *Phys. Rev. Lett.* **110**, 034101 (2013).
32. Chen, L., Kottos, T. & Anlage, S. M. Perfect absorption in complex scattering systems with or without hidden symmetries. *Nat. Commun.* **11**, 5826 (2020).
33. Peatman, W. C. B., Wood, P. A. D., Porterfield, D., Crowe, T. W. & Rooks, M. J. Quarter-micrometer GaAs Schottky barrier diode with high video responsivity at 118 μm. *Appl. Phys. Lett.* **61**, 294–296 (1992).
34. Barker, A. S., Verleur, H. W. & Guggenheim, H. J. Infrared Optical Properties of Vanadium Dioxide Above and Below the Transition Temperature. *Phys. Rev. Lett.* **17**, 1286–1289 (1966).
35. Ghanekar, A., Ji, J. & Zheng, Y. High-rectification near-field thermal diode using phase change periodic nanostructure. *Appl. Phys. Lett.* **109**, 123106 (2016).
36. Li, L. *et al.* Electromagnetic reprogrammable coding-metasurface holograms. *Nat. Commun.* **8**, 197 (2017).
37. Stolyarov, A. M. *et al.* Fabrication and characterization of fibers with built-in liquid crystal channels

and electrodes for transverse incident-light modulation. *Appl. Phys. Lett.* **101**, 011108 (2012).
38. Gholipour, B., Zhang, J., MacDonald, K. F., Hewak, D. W. & Zheludev, N. I. An All-Optical, Non-volatile, Bidirectional, Phase-Change Meta-Switch. *Adv. Mater.* **25**, 3050–3054 (2013).
39. Wang, D. *et al.* Switchable Ultrathin Quarter-wave Plate in Terahertz Using Active Phase-change Metasurface. *Sci. Rep.* **5**, 15020 (2015).
40. Wang, Q. *et al.* Optically reconfigurable metasurfaces and photonic devices based on phase change materials. *Nat. Photonics* **10**, 60–65 (2016).
41. Ju, L. *et al.* Graphene plasmonics for tunable terahertz metamaterials. *Nat. Nanotechnol.* **6**, 630–634 (2011).
42. Yao, Y. *et al.* Broad Electrical Tuning of Graphene-Loaded Plasmonic Antennas. *Nano Lett.* **13**, 1257–1264 (2013).
43. Huang, Y.-W. *et al.* Gate-Tunable Conducting Oxide Metasurfaces. *Nano Lett.* **16**, 5319–5325 (2016).
44. Bruck, R. *et al.* All-optical spatial light modulator for reconfigurable silicon photonic circuits. *Optica* **3**, 396 (2016).
45. Bello-Jiménez, M. *et al.* Actively mode-locked all-fiber laser by 5 MHz transmittance modulation of an acousto-optic tunable bandpass filter. *Laser Phys. Lett.* **15**, 085113 (2018).
46. Matthès, M. W., del Hougne, P., de Rosny, J., Lerosey, G. & Popoff, S. M. Optical complex media as universal reconfigurable linear operators. *Optica* **6**, 465 (2019).
47. Ou, J. Y., Plum, E., Jiang, L. & Zheludev, N. I. Reconfigurable Photonic Metamaterials. *Nano Lett.* **11**, 2142–2144 (2011).
48. Ou, J.-Y., Plum, E., Zhang, J. & Zheludev, N. I. An electromechanically reconfigurable plasmonic metamaterial operating in the near-infrared. *Nat. Nanotechnol.* **8**, 252–255 (2013).
49. Resisi, S., Viernik, Y., Popoff, S. M. & Bromberg, Y. Wavefront shaping in multimode fibers by transmission matrix engineering. *APL Photonics* **5**, 036103 (2020).
50. Chen, X. *et al.* Active acoustic metamaterials with tunable effective mass density by gradient magnetic fields. *Appl. Phys. Lett.* **105**, 071913 (2014).
51. Xiao, S., Ma, G., Li, Y., Yang, Z. & Sheng, P. Active control of membrane-type acoustic metamaterial by electric field. *Appl. Phys. Lett.* **106**, 091904 (2015).
52. Chen, Z. *et al.* A tunable acoustic metamaterial with double-negativity driven by electromagnets. *Sci. Rep.* **6**, 30254 (2016).
53. Ma, G., Fan, X., Sheng, P. & Fink, M. Shaping reverberating sound fields with an actively tunable metasurface. *Proc. Natl. Acad. Sci. USA* **115**, 6638–6643 (2018).
54. Ao, W., Ding, J., Fan, L. & Zhang, S. A robust actively-tunable perfect sound absorber. *Appl. Phys. Lett.* **115**, 193506 (2019).
55. Tian, Z. *et al.* Programmable Acoustic Metasurfaces. *Adv. Funct. Mater.* **29**, 1808489 (2019).
56. Cao, W. K. *et al.* Tunable Acoustic Metasurface for Three-Dimensional Wave Manipulations. *Phys. Rev. Applied* **15**, 024026 (2021).
57. Zhang, C. *et al.* A reconfigurable active acoustic metalens. *Appl. Phys. Lett.* **118**, 133502 (2021).
58. Zhao, S.-D., Chen, A.-L., Wang, Y.-S. & Zhang, C. Continuously Tunable Acoustic Metasurface for Transmitted Wavefront Modulation. *Phys. Rev. Applied* **10**, 054066 (2018).
59. Fan, S.-W. *et al.* Tunable Broadband Reflective Acoustic Metasurface. *Phys. Rev. Applied* **11**,

044038 (2019).

60. Ma, Z. *et al.* Spatial ultrasound modulation by digitally controlling microbubble arrays. *Nat. Commun.* **11**, 4537 (2020).
